# Cognitive function in UK adults seropositive for *Helicobacter pylori*

**DOI:** 10.1371/journal.pone.0286731

**Published:** 2023-06-07

**Authors:** Lance D. Erickson, David S. White, Pierce Bassett, Shawn D. Gale, Bruce L. Brown, Dawson Hedges

**Affiliations:** 1 Department of Sociology, Brigham Young University, Provo, Utah; 2 Department of Microbiology and Molecular Biology, Brigham Young University, Provo, Utah; 3 The Neuroscience Center, Brigham Young University, Provo, Utah; 4 Department of Psychology, Brigham Young University, Provo, Utah; University of Catania Department of Surgical and Medical Sciences Advanced Technologies GF Ingrassia: Universita degli Studi di Catania Dipartimento di Scienze Mediche Chirurgiche e Tecnologie Avanzate GF Ingrassia, ITALY

## Abstract

Associated with gastritis, peptic-ulcer disease, and gastric carcinoma, *Helicobacter pylori* (*H*. *pylori*) also has been associated with decreased cognitive function and dementia. In this study, we used data from the UK Biobank to further examine associations between *H*. *pylori* seropositivity and serointensity and performance on several cognitive tasks in adults 40 to 70 years of age (M = 55.3, SD = 8.1). In these analyses, *H*. *pylori* seropositivity (i.e., either positive or negative for *H*. *pylori*) and serointensity (concentration of antibodies against *H*. *pylori* antigens) in adjusted models were associated with worse function on tasks of Numeric memory, Reasoning, and errors on the Pairs matching test but better function on the Tower rearrangement task. Together, these findings suggest that *H*. *pylori* seropositivity and serointensity might be associated with worse cognitive function in this age group.

## Introduction

*Helicobacter pylori* (*H*. *Pylori*) is a gram-negative bacterium that infects the stomach and duodenum in humans [1]. Most commonly, infection with *H*. *pylori* occurs during childhood, with characteristics of infection ranging from asymptomatic to mild (e.g., gastric distress, abdominal pain) to severe (e.g., peptic-ulcer disease, atrophic gastritis, gastric carcinoma) [2–5]. *H*. *pylori* infection occurs most commonly through ingestion, although transmission can also occur from mother to fetus [2]. *H*. *pylori* is estimated to infect more than one-half of the human population [6]. Despite host-mediated immune response to *H*. *Pylori*, infection can persist for the lifetime of the host in the absence of antibacterial treatment [7,8].

In addition to its associations with gastric disease, *H*. *pylori* seropositivity has been associated with decreased cognitive function in some, but not all, studies. In a study of US adults using data from the US Centers for Disease Control and Prevention’s National Health and Examination Surveys (NHANES), *H*. *pylori* was adversely associated with cognitive function [9]. In contrast, a study of young to middle-aged adults (20 to 59 years of age) that also used data from the NHANES found no main effects between *H*. *pylori* and cognitive function [10], although statistically significant interactions between *H*. *pylori* seropositivity and both race-ethnicity and educational attainment predicted cognitive function. Furthermore, the study found an interaction between *H*. *pylori* seropositivity and a common parasitic infection, *Toxoplasma gondii*, such that the presence of both was associated with decreased cognitive function. Using data from the NHANES, Cárdenas et al. [11], however, found that older adults seropositive for *H*. *Pylori* had depressed scores on the digit-symbol substitution test. In addition, *H*. *pylori* has been associated with dementia [12,13].

Infection with *H*. *pylori* is common, and an association between *H*. *pylori* and cognitive function would represent both personal and public-health problems. However, *H*. *pylori* is potentially treatable. In that some early reports show possible associations between *H*. *pylori* and decreased cognitive function [9–11] and because of possible associations between *H*. *pylori* and dementia [12,13], we sought to further characterize the relationship between *H*. *pylori* seropositivity and serointensity and cognitive function.

## Materials and methods

### Study sample

We obtained data from the UK Biobank Resource, a community-based sample of adults for which the National Research Ethics Service Committee North West-Haydock provided approval (reference 11/NW/0382). All participants provided informed consent (http://biobank.ctsu.ox.ac.uk/crystal/field.cgi?id=200). Data can be obtained through application to the UK Biobank http://www.ukbiobank.ac.uk. We received approval to use deidentified data under application number 41535. Although the UK Biobank data are not representative of the UK population and the UK Biobank had a low initial response rate, research findings suggest that data from the UK Biobank can be used to investigate exposure and outcome associations [14]. The UK Biobank enrolled approximately 500,000 adults from ages 40 to 70 years from population-based registries (http://www.ukbiobank.ac.uk) and obtained clinical and demographic data from biological samples, nurse interviews, physical examinations, and questionnaires (http://biobank.ctsu.ox.ac.uk/crystal/field.cgi?id=200).

The analytic sample we used included all participants who had seropositivity and serointensity data for *H*. *pylori*, had undergone testing of cognitive function, and who had the preidentified covariates. Sample sizes varied across the individual tests of cognitive function based on availability of the relevant data (https://biobank.ctsu.ox.ac.uk/crystal/crystal/docs/infdisease.pdf), ranging from 379 to 6,785.

### Measures

#### H. pylori

In the UK Biobank, *H*. *pylori* seropositivity was defined based on concentrations of antibodies against CagA, VacA, OMP, GroEL, Catalase, and UreA antigens. Because of a lab-handling error, there are two definitions of *H*. *pylori* seropositivity in these data. In the first definition, participants were considered seropositive if they were positive for two of the six antibodies measured using median fluorescence intensity (MFI). Cutoffs were as follows: 400 for CagA, 100 for VacA, 170 for OMP, 80 for GroEL, 180 for Catalase, and 130 for UreA. The second definition also required positive tests for two of the antigens using these cutoffs but excluded the CagA antigen because it was the object of the lab error. In addition to using seropositivity, we also used natural-log (ln) transformed concentrations of the CagA, VacA, OMP, GroEL, Catalase, and UreA antibodies as measures of serointensity. Finally, we standardized the logged versions of the antigens and included their mean. For participants who did not have a measure of the CagA antigen, we excluded that from this measure (https://biobank.ctsu.ox.ac.uk/crystal/field.cgi?id=23062).

#### Cognitive function

The UK Biobank assessed cognitive function with a battery of neuropsychological tasks. Further descriptions of these cognitive tasks can be found elsewhere: https://biobank.ctsu.ox.ac.uk/crystal/label.cgi?id=100026. Briefly, Numeric memory, a measure of working memory, requires participants to view an increasing longer string of numbers and then after a brief delay to write what they saw (higher score is better). Reasoning consists of questions that require logic, executive function, and problem-solving skills (higher score is better). Pairs matching, a memory task, requires memorization and recall of the location of similar cards. There were six pairs and participants were allowed to continue until they matched all six pairs correctly, which nearly all did. Thus, due to a ceiling effect, we analyzed the number of errors (Pairs matching incorrect; lower score is better). Matrix pattern completion, a visual pattern reasoning task, requires participants to view a pattern with a missing piece and select one that would complete the pattern (higher score is better). Tower rearrangement, an executive functioning task, includes planning a sequence of moves and rule-following to rearrange the stimuli into directed configurations (higher score is better). Symbol-digit substitution, which includes elements of processing speed, psychomotor function, and memory, consists of matching symbols and digits per a guide as quickly as possible during a time limit (correct matches; higher score is better). Reaction time measures average response time (in milliseconds) to correctly identify matching versus non-matching image pairs (less total time is better). Give the skewness of the data from the Reaction time test, we analyzed the natural log of these scores on this test. Trails: numeric assesses total time to click sequentially on set of numbers on the screen (less total time is better). Trails: numeric measures psychomotor speed and visual attention. Trails: alphanumeric is similar except there are both numbers and letters on the screen and the participant must click on them sequentially alternating between numbers and letters (i.e., 1, A, 2, B etc.). Performance is based on total time to complete the task (less total time is better). Trails: alphanumeric requires skills similar to those required on Trails: numeric but additionally requires an element of executive function. Information regarding reliability and validity of the UK Biobank cognitive tests can be found elsewhere [15].

#### Covariates

To control for variables that potentially could confound associations between *H*. *pylori* infection and cognitive function, we adjusted all statistical models for age in years, sex (female, male), race-ethnicity (White, non-White), income (the midpoint of reported categories in 10,000 pounds/year: less than 18,000; 18,000 to 30,999; 31,000 to 51,999; 52,000 to 99,999; and 100,000£ and above), educational attainment (college degree, less than college degree), self-rated health (four-point scale from poor to excellent), body-mass index (body weight in kg/height in m^2^), smoking history (non-smoker, past, current), and alcohol use (six categories ranging from never to daily or almost daily).

### Statistical analysis

Using Stata 17.0 (StataCorp, Stata Statistical Software, Release 17.0. College Station, Texas) for all statistical analyses, we first estimated a series of adjusted multivariable linear regression models using the nine measures of *H*. *pylori* as focal independent variables in separate models of each of the nine cognitive tests for a total of 72 models. We also estimated adjusted interaction models of age, sex, educational attainment, and income with each measure of *H*. *pylori* for each of the nine cognitive functioning measures. This is an additional 72 models for each of the four interaction models, or an additional 288 models.

With such a large number of hypothesis tests, we estimated multivariate tests using Stata’s *suest* command to decrease the risk of false-positive results from multiple comparisons. The *suest* command combined model estimates of a single predictor (e.g., *H*. *pylori* seropositivity) and the nine cognitive functioning dependent variables into a single parameter vector. The resulting vector accounts for the joint covariance structure of the outcome variables. Then, rather than traditional estimates of multivariate significance, such as Wilk’s Lambda, Pillai’s Trace, Hotelling-Lawley Tract, and Roy’s Greatest Root [16], a single multivariate test is estimated with the null hypothesis that the joint relationship between the predictor and each of the outcomes is zero. If the *p* value from the multivariate test was not statistically significant (that is, if the multivariate *p* value was greater than .05), we did not consider any associations between the focal predictor variable and an outcome variable to be statistically significant in the individual models. This is in accordance with the recommendations from Rencher and Scott’s multivariate simulation study [16,17]. Like the four traditional multivariate tests of significance, the *suest* command method accounts for the covariance structure of the multivariate space. This strikes a balance between protecting against alpha inflation while maintaining statistical power [16,18].

## Results

Table 1 shows the demographic characteristics of the sample. The analytic sample size varied depending on the data available for the cognitive functioning variable, ranging from 379 to 6,785. In the largest analytic sample, 30.1 percent were *H*. *pylori* seropositive. The average age was 55.3 years, and women comprised 54.6% of the sample. The sample was predominantly white (95.1%), and 40.1% had obtained a college degree.

**Table 1:** Descriptive statistics of study variables.

	Mean	SD	Minimum	Maximum	N
Cognition					
Numeric memory	6.77	1.31	2	12	796
Reasoning	6.36	2.09	0	13	2,269
Pairs matching incorrect	4.10	3.35	0	39	6,785
Matrix pattern completion	8.29	2.00	2	14	393
Tower rearrangement	10.31	3.65	0	18	398
Symbol-digit substitution	19.54	5.11	2	36	395
Reaction time	547.71	108.55	320	1594	6,757
Trails: Numeric	221.43	89.37	107	1098	394
Trails: Alphanumeric	563.20	272.85	244	2442	379
*H*. *Pylori*					
Seropositive^a^	.30		0	1	6,785
ln(CagA)	4.55	2.02	0	9	3,410
ln(VacA)	3.42	1.56	0	9	6,785
ln(OMP)	4.04	2.12	0	9	6,785
ln(GroEL)	3.53	2.70	0	9	6,785
ln(Catalase)	3.95	1.76	0	10	6,785
ln(UreA)	3.62	2.12	0	10	6,785
Mean of standardized antigens	-.05	.71	-2	2	6,785
Age	55.30	8.13	40	70	6,785
Female	.55		0	1	6,785
White	.95		0	1	6,785
College degree	.41		0	1	6,785
Income (in 10,000£)	4.50	3.09	1	12	6,785
Self-rated health	2.93	.73	1	4	6,785
Body-mass index	27.16	4.73	16	61	6,785
Smoking status					
Non-smoker	.58		0	1	6,785
Past	.33		0	1	6,785
Current	.09		0	1	6,785
Drinking frequency					
Daily or almost daily	.23		0	1	6,785
3–4 times/week	.24		0	1	6,785
Once or twice/week	.25		0	1	6,785
1–3 times/month	.11		0	1	6,785
Special occasions	.11		0	1	6,785
Never	.06		0	1	6,785

Note

^a^ Proportion reported. Source: *UK Biobank*.

Adjusted models of the various *H*. *pylori* measures and their relationship with cognitive functioning are presented in Table 2. *H*. *pylori* seropositivity was associated with worse performance on the Reasoning (b = -.386, *p* < .001) and Pairs matching incorrect (b = .238, *p* < .01) tasks but with better performance on Tower rearrangement (b = 1.346, *p* < .001) task; the associated *p* value from the multivariate test was < .001. lnCagA was associated with worse performance on Numeric memory (b = -.087, *p* < .01) and Reasoning (b = -073, *p* < .05) tasks; the *p* value from the multivariate test was .002. lnVacA was associated with worse performance on the Reasoning task (b = -.079, *p* < .01); the *p* value from the multivariate test was .048. lnOMP was associated with worse Reasoning (b = -.080, *p* < .001); the *p* value from multivariate test was < .001). lnGroEL was associated with worse Reasoning (b = -.066, *p* < .001) but with better performance on Tower rearrangement (b = .135, *p* < .05); the *p* value from the multivariable test was .001). lnCatalase was associated with worse Reasoning (b = -.081, *p* < .001), but the associated *p* value from the multivariate test was only marginally significant at .051. lnUreA was associated with worse Reasoning (b = -.059, *p* < .01); the *p* value from the associated multivariate test was .049. The mean of the standardized antigens was associated with worse Reasoning (b = -.296, *p* < .001) and with better performance on the Tower rearrangement task (b = .516, *p* < .05); the *p* value from the associated multivariate test was < .001 (Table 2).

**Table 2:** Adjusted models of the effects of *H*. *Pylori* on nine measures of cognitive functioning: Unstandardized coefficients from linear regression.

	Seropositive	ln(CagA)	ln(VacA)	ln(OMP)	ln(GroEL)	ln(Catalase)	ln(UreA)	Mean of standardized antigens
Cognitive functioning								
Numeric memory	-.167	-.087**	-.019	-.021	-.014	-.033	-.004	-.085
Reasoning	-.386***	-.073*	-.079**	-.080***	-.066***	-.081***	-.059**	-.296***
Pairs matching incorrect	.238**	-.035	.012	.021	.025	.017	-.004	.047
Matrix pattern completion	.166	-.009	-.022	-.033	-.009	.038	.040	-.002
Tower rearrangement	1.346***	.142	.192	.085	.135*	.136	.094	.516*
Symbol-digit substitution	-.048	.100	.155	-.054	.027	-.008	-.080	.048
Reaction time	.359	.601	.114	-.342	-.070	.029	.933	.573
Trails: Numeric	-6.463	.135	.685	-.673	.153	.737	1.695	1.962
Trails: Alphanumeric	-32.546	-19.428	-5.541	-11.038	-1.415	-1.144	-4.626	-22.928
Multivariate test^a^								
*p*	< .001	.002	.048	< .001	.001	.051	.049	< .001

Note: Each cell in the table represents the results from a separate model. The main independent variable is listed in the column headers and the dependent variable is listed in the row labels. Each model is adjusted for age, sex, white, college degree, household income, self-rated health, body-mass index, smoking status, and frequency of drinking alcohol.

^a^ The multivariate test is a holistic test of the null hypothesis considered within the multivariate space defined by the nine cognitive function. This is shown in each column of the table for a particular one of the seven measures of *H*. *pylori* (i.e., *H*. *pylori* seropositive; the natural log of CagA; VacA; OMP; GroEL; Catalase; UreA; and the mean of standardized antigens). It is applied here to address potential problems of reporting false positives because of the number of statistical tests performed. Significant relationships between an *H*. *pylori* measure and cognitive function are thus ignored if the probability of the multivariate null being true is greater than .05. Numeric N = 796, Reaction time N = 6,757, Reasoning N = 2,269, Pairs matching N = 6,785, Trails numeric N = 394, Trails alphanumeric N = 379, Matrix pattern completion N = 393, Tower rearrangement N = 398, Symbol-digit substitution N = 395.

* *p* < .05

** *p* < .01

*** *p* < .001.

Source: *UK Biobank*.

**Fig 1:**
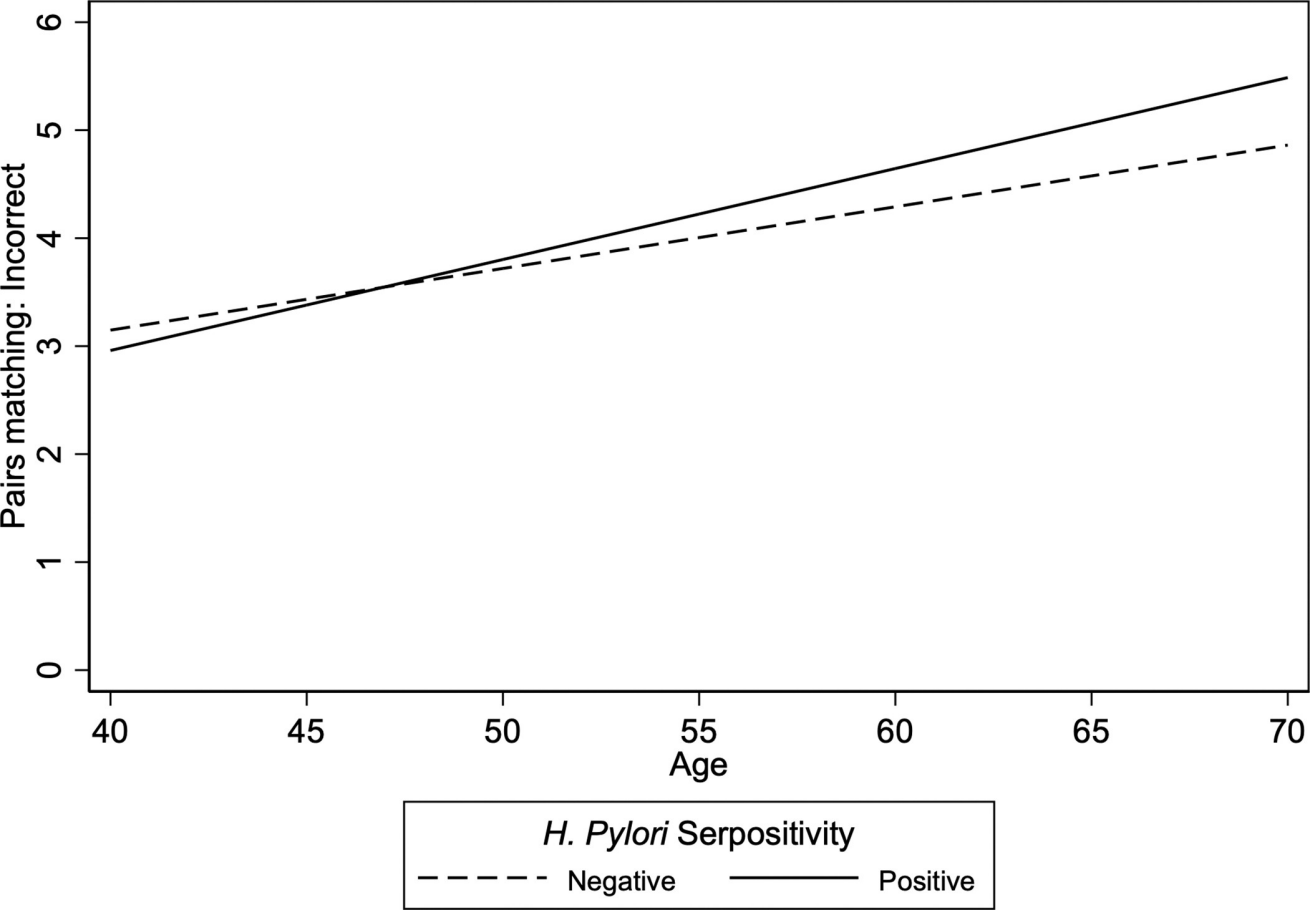
Interaction of *H*. *Pylori* Seropositivity and age on Pairs Matching: Incorrect Adjusted predictions from linear regression. Note: N = 6785. ^a^ Models adjusted for sex, race, education, household income, self-rated health, body-mass index, smoking status, and frequency of drinking alcohol. Source: UK Biobank.

Of 32 multivariate models of interactions between *H*. *pylori* and age, sex, educational attainment, and income in predicting cognitive function, only three were statistically significant: *H*. *pylori* seropositivity and age (*p* = .019), ln(CagA) and age (*p* = .032), and ln(GroEL) and sex (*p* = .027) (Table 3). In these models, *H*. *pylori* seropositivity was associated with worse performance on the Pairs matching incorrect task with increasing age (S1 Table in S1 File, Fig 1) but with worse performance on the Trails numeric task with increasing age (S1 Table in S1 File, S1 Fig) compared to *H*. *pylori* seropositivity. ln(CagA) negative was associated with worse Trails numeric function with increasing age compared to ln(CagA) positive (S1 Table in S1 File, S2 Fig). Similarly, ln(CagA) negative was associated with worse Trails alphanumeric function with increasing age compared to ln(CagA) positive groups (S1 Table in S1 File, S3 Fig). Increasing ln(GroEL) decreased differences between women and men in the Tower rearrangement task (S2 Table in S1 File, S4 Fig). There were no multivariately statistically significant interactions with either educational attainment or income (S3 and S4 Tables in S1 File).

**Table 3:** Multivariate *P* values from adjusted models cognitive functioning including interactions of *H*. *pylori* with age, sex, education, and income.

	Seropositivity	ln(CagA)	ln(VacA)	ln(OMP)	ln(GroEL)	ln(Catalase)	ln(UreA)	Mean of standardized antigens
*H*. *pylori* x Age	.019	.032	.124	.266	.473	.418	.063	.107
*H*. *pylori* x Female	.607	.787	.692	.594	.027	.865	.432	.371
*H*. *pylori* x College degree	.200	.711	.218	.238	.095	.418	.108	.097
*H*. *pylori* x Income	.562	.091	.219	.281	.308	.148	.672	.614

Note: Each cell in the table represents the *P* value for the holistic multivariate pattern of each interaction in the multivariate space defined by the nine cognitive functioning dependent variables. The column header represents the *H*. *pylori* measure included in the interaction. The object of the multivariate test is the interaction listed in the row labels. For example, the intersection of the first row and column is the multivariate *P* value for the test of whether there is an interaction between *H*. *pylori* seropositivity and age that is jointly related to the nine cognitive functioning dependent variables. Individual models were adjusted for age, sex, white, college degree, household income, self-rated health, body-mass index, smoking status, and frequency of drinking alcohol. Results of the models that are represented in these multivariate tests are presented in supplemental tables. Source: *UK Biobank*.

## Discussion

In this sample from the UK Biobank, *H*. *pylori* seropositivity and *H*. *pylori* serointensity assessed by natural log-transformed concentrations of the *H*. *pylori* CagA, VacA, OMP, GroEL, and UreA antigens were negatively associated as a group with performance on the Numeric memory, Reasoning, and Pairs matching incorrect tasks, with better performance on the Tower rearrangement task, and with no associations with the Matrix pattern-completion, Symbol-digit substitution, Reaction-time, Trails-numeric, and Trails-alphanumeric tasks in adjusted models constrained by multivariate testing to protect against false positive from multiple comparisons. The only *H*. *pylori* antigen not statistically significantly associated with cognitive function was Catalase. Worse performance on the Reasoning task was the most consistently associated with *H*. *pylori*. There were fewer negative associations with performance on the Numeric memory and Pairs-matching incorrect tasks and none with performance on the Symbol-digit substitution, Trails numeric and Trails alphanumeric tasks. Apart from better performance on the Tower rearrangement task, this pattern suggests that executive function might be particularly vulnerable to infection with *H*. *pylori*, although we were able to examine associations between *H*. *pylori* and cognitive function in only nine tasks of cognitive function. Had more tasks including ones from other cognitive domains been available, it is possible that other patterns of potential vulnerability would have been found.

These associations between *H*. *pylori* seropositivity and serointensity add to previous findings showing associations between *H*. *pylori* and worse cognitive function [9–11] but in a sample from the United Kingdom instead of the United States. However, our results differ from a previous study that did not find associations between *H*. *pylori* seropositivity and performance on the digit-symbol coding task in older US adults, although this study did find that *H*. *pylori* seropositivity did interact with 5-methytetrahydrofolate concentrations to affect performance on the digit-symbol coding task [19], a finding that suggests a possible association between *H*. *pylori* infection and worse cognitive function.

While there were few significant findings from the interaction models, findings from models constrained by multivariate analyses to protect against false-positive results from multiple comparisons suggest that age might interact with *H*. *pylori* to affect cognitive function. The findings from these few statistically significant interaction models showed mixed results. With overall seropositivity and function on the Pairs matching incorrect task, *H*. *pylori* seropositivity was associated with worse function with increased age, which suggests the possibility that *H*. *pylori* could be a factor in the development of dementia consistent with findings showing possible associations between *H*. *pylori* and dementia [12,13]. However, the other statistically significant interaction models showed better function on Trails: numeric and Trails: alphanumeric task with increased antibody titers and age, findings suggesting a more nuanced association between *H*. *pylori* and cognitive function at least on the Trails tasks with age.

If replicated, however, the associations we found between *H*. *pylori* and decreased cognitive function suggest that *H*. *pylori* infection could be a clinically significant risk factor for cognitive dysfunction and possibly even dementia, particularly given the widespread distribution of infection with *H*. *pylori* [6]. Available treatment for *H*. *pylori* makes *H*. *pylori* infection a potentially modifiable risk factor for cognitive decline. In this regard, treatment for *H*. *pylori* decreased mortality in patients with Alzheimer disease [20], although to our knowledge no studies to date have evaluated whether treatment of *H*. *pylori* infection improves cognitive function or affects the incidence of dementia.

We did not design our study to investigate mechanisms by which *H*. *pylori* could affect cognitive function. Work from previous studies, however, has identified several potential mechanisms. Emerging evidence indicates that the composition of and disturbances of the gut microbiome can be associated with cognitive function [21]. In this regard, *H*. *pylori* colonization in the stomach and duodenum could feasibly alter the gut microbiome and bidirectional signaling with the brain to affect cognitive function. *H*. *pylori* infection can result in decreased vitamin B_12_ and folate absorption, which in turn has been associated with elevated concentrations of homocysteine, which have been associated with atherosclerosis [5], decreased cognitive function, and dementia [22]. In addition, altered 5-methyltetrahydrofolate [19] and folate metabolism and inflammatory markers could mediate associations between *H*. *pylori* infection and cognitive function [23]. Other possible mechanisms by which *H*. *pylori* could affect brain function leading to worse cognitive function could be due to T cell-mediated apoptosis, molecular mimicry of host structures [24], induced inflammation from influx of cytokines, platelets, acute phase proteins, or eicosanoids, or oxidative stress [25]. As *H*. *pylori* infection can possibly disrupt the blood-brain barrier via inflammatory factors and oxidative stress, additional causes of decreased cognitive function from *H*. *pylori infection* could be related to amyloid deposition induced by pathogens passing through a disrupted blood brain barrier [20]. Similarly, in rats, *H*. *pylori* itself might increase hippocampal and cortical amyloid 42 via increased gamma secretase activity [26], suggesting that *H*. *pylori* could also affect cognition via increased amyloid deposition. These putative mechanisms by which *H*. *pylori* might affect cognitive function provide additional potential avenues for treatment to mitigate against the effects of *H*. *pylori* on cognitive function.

While our study was large with sample sizes ranging from 379 to 6,785 and included analyses adjusted for several covariates that could confound associations between *H*. *pylori* and cognitive function, several factors require consideration when interpreting these findings. The study design was cross-sectional, precluding the study’s ability to determine the temporal ordering of *H*. *pylori* and cognitive function. Being unable to determine when the initial infection with *H*. *pylori* occurred, we could not address whether infection at different neurodevelopmental stages could affect the relationship between *H*. *pylori* infection and cognitive function. An important limitation of the existing research of the associations between *H*. *pylori* infection and cognitive function is that most of the findings come from samples in either the United Kingdom or the United States. Findings from samples from other regions are needed to better characterize associations between *H*. *pylori* and cognitive function. While we adjusted for nine variables that could potentially alter the association between *H*. *pylori* and cognitive function, numerous factors are associated with cognitive outcomes, leading to the possibility of residual confounding. An additional limitation of our study is that we did not adjust the statistical models for use of gastroprotective drugs, including proton-pump inhibitors, which could affect the association we found between *H*. *pylori* and cognitive function. While a study based on UK Biobank data found an association between use of proton-pump inhibitors and dementia, with the association being strongest in carriers of the apolipoprotein E4 allele [27], three meta-analyses [28–30] and a study with longitudinal neuropsychological testing [31] did not show an association between proton-pump inhibitors and dementia. Given these mixed findings, future studies regarding associations between *H*. *pylori* and cognitive function may want to consider including controlling for use of proton-pump inhibitors. Finally, our results do not address whether treatment for *H*. *pylori* would result in any changes in cognition.

In conclusion, the results from this study show that *H*. *pylori* seropositivity and serointensity are associated with worse function on tasks of Numeric memory, Reasoning, and Pairs matching incorrect but with better performance on the Tower rearrangement task. Further, research investigating whether potentially confounding variables such as the use of proton-pump inhibitors might affect the association between *H*. *pylori* and cognitive function is needed. Additional future research regarding associations between *H*. *pylori* and cognitive function from regions outside of the United Kingdom and the United States is needed as is research using longitudinal studies, possibly by using data from follow-up assessments in the UK Biobank and by linking datasets where one contains exposure data and a second, later dataset has some sort of cognitive outcome variable, including dementia.

## Supporting information

S1 Fig Interaction of H. Pylori and age on Trails: Numeric: Adjusting predictions^a^ from linear regression. Note: N = 394. ^a^Models adjusted for sex, race, education, household income, self-rated health, body-mass index, smoking status, and frequency of drinking alcohol. Source: UK Biobank.
(TIF)

S2 Fig Interaction of ln(CagA) and age on Trails: Numeric: Adjusting predictions^a^ from linear regression. Note: N = 213. ^a^Models adjusted for sex, race, education, household income, self-rated health, body-mass index, smoking status, and frequency of drinking alcohol. Source: UK Biobank. ln(CagA) = natural log of CagA antibody concentration.
(TIF)

S3 Fig Interaction of ln(CagA) and age on Trails: Alphanumeric: Adjusting predictions^a^ from linear regression. Note: N = 204. ^a^Models adjusted for sex, race, education, household income, self-rated health, body-mass index, smoking status, and frequency of drinking alcohol. Source: UK Biobank. ln(CagA) = natural log of CagA antibody concentration.
(TIF)

S4 Fig Interaction of ln(CagA) and age on Tower Rearrangement: Adjusting predictions^a^ from linear regression. Note: N = 398. ^a^Models adjusted for sex, race, education, household income, self-rated health, body-mass index, smoking status, and frequency of drinking alcohol. Source: UK Biobank. ln(GroEL) = natural log of GroEL antibody concentration. ln(CagA) = natural log of CagA antibody concentration.
(TIF)

S1 File Supplemental tables. (PDF)
