# Supplementary figures and images for: Cognitive function in UK adults seropositive for Helicobacter pylori
Source: PLoS One. 2023 Jun 7;18(6):e0286731. doi: 10.1371/journal.pone.0286731 (PMC10246820; doi:10.1371/journal.pone.0286731)

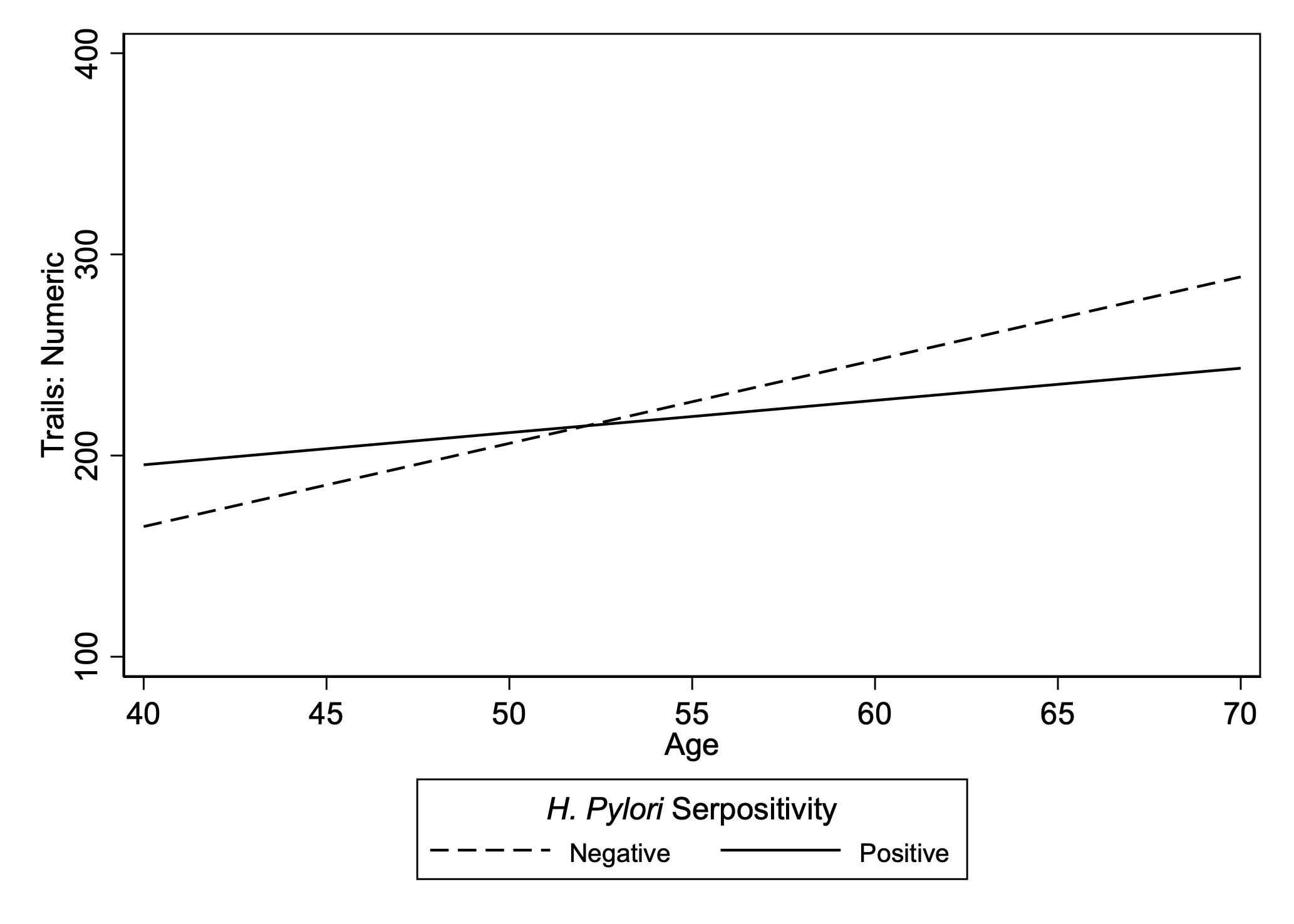

Supplement: S1 Fig — Note: N = 394. aModels adjusted for sex, race, education, household income, self-rated health, body-mass index, smoking status, and frequency of drinking alcohol. Source: UK Biobank. (TIF) [file pone.0286731.s001.tif]

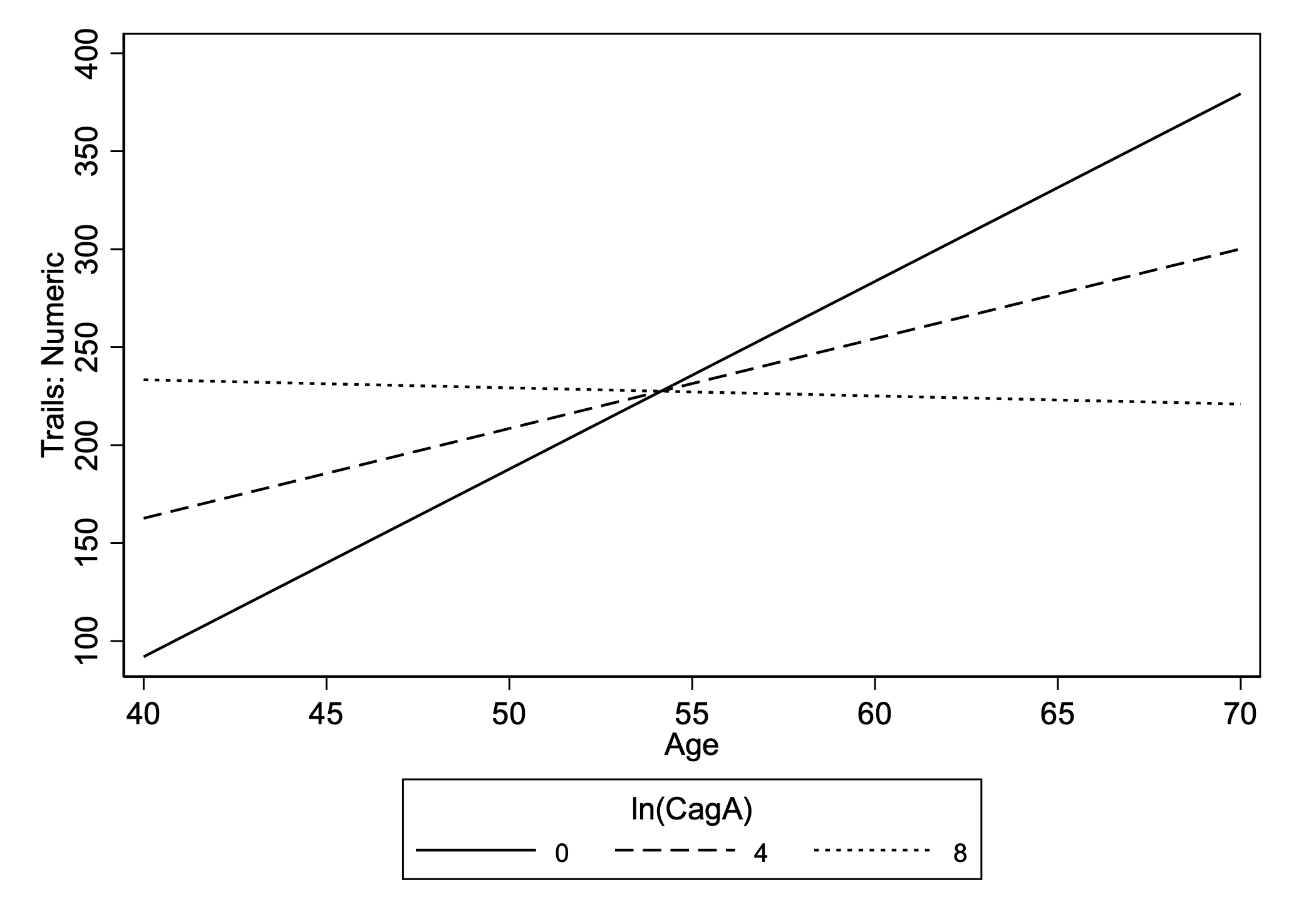

Supplement: S2 Fig — Note: N = 213. aModels adjusted for sex, race, education, household income, self-rated health, body-mass index, smoking status, and frequency of drinking alcohol. Source: UK Biobank. ln(CagA) = natural log of CagA antibody concentration. (TIF) [file pone.0286731.s002.tif]

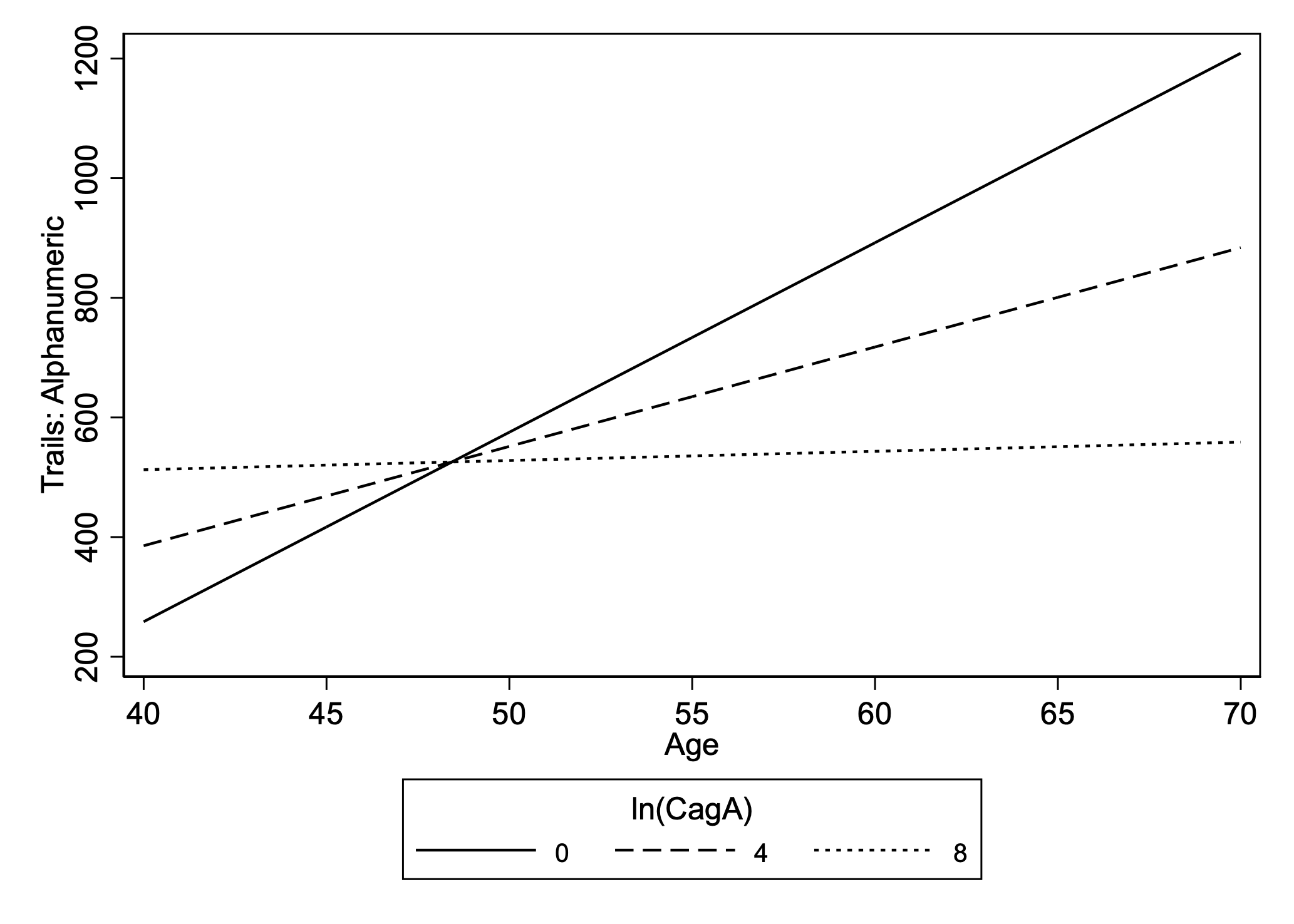

Supplement: S3 Fig — Note: N = 204. aModels adjusted for sex, race, education, household income, self-rated health, body-mass index, smoking status, and frequency of drinking alcohol. Source: UK Biobank. ln(CagA) = natural log of CagA antibody concentration. (TIF) [file pone.0286731.s003.tif]

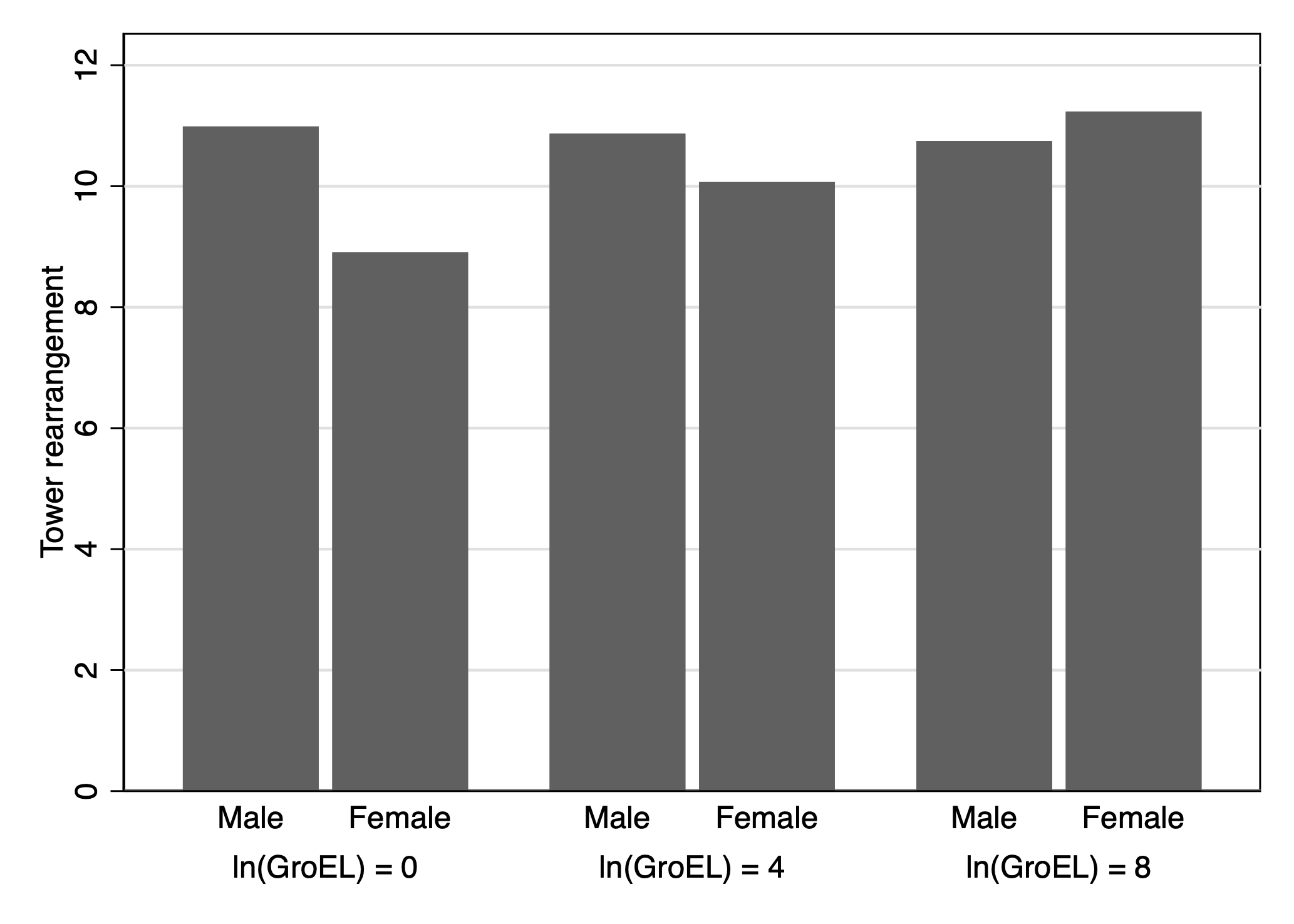

Supplement: S4 Fig — Note: N = 398. aModels adjusted for sex, race, education, household income, self-rated health, body-mass index, smoking status, and frequency of drinking alcohol. Source: UK Biobank. ln(GroEL) = natural log of GroEL antibody concentration. ln(CagA) = natural log of CagA antibody concentration. (TIF) [file pone.0286731.s004.tif]
