# Supplementary material for: Cognitive function in UK adults seropositive for Helicobacter pylori
Source: PLoS One. 2023 Jun 7;18(6):e0286731. doi: 10.1371/journal.pone.0286731 (PMC10246820; doi:10.1371/journal.pone.0286731)
Supplement: S1 File — (PDF) [file pone.0286731.s005.pdf]

Supplemental Table 1.
Adjusted models of cognitive functioning on the interaction of *H. pylori* and age: Unstandardized coefficients from linear regression

	Seropositive	ln(CagA)	ln(VacA)	ln(OMP)	ln(GroEL)	ln(Catalase)	ln(UreA)	Mean of standardized antigens
Numeric memory								
H. Pylori	-1.026	-.349	-.233	-.191	-.019	-.067	.041	-.426
Age	-.008	-.013	-.017	-.017	-.005	-.007	-.002	-.004
H. Pylori x Age	.015	.005	.004	.003	.000	.001	-.001	.006
Reasoning								
H. Pylori	-1.452*	-.206	-.516**	-.239	-.079	-.171	.004	-.852*
Age	-.004	-.012	-.025*	-.010	.002	-.005	.005	.002
H. Pylori x Age	.019	.002	.008*	.003	.000	.002	-.001	.010
Pairs matching incorrect								
H. Pylori	-1.274*	-.144	.185	.180	-.211*	.048	.047	.092
Age	.057***	.060***	.077***	.077***	.051***	.068***	.069***	.066***
H. Pylori x Age	.027*	.002	-.003	-.003	.004*	-.001	-.001	-.001
Matrix pattern completion								
H. Pylori	-1.926	-.600	-.674	-.095	.014	-.562	-.324	-1.599
Age	-.062***	-.100*	-.090**	-.055	-.048*	-.093**	-.073**	-.047**
H. Pylori x Age	.038	.011	.012	.001	-.000	.011	.007	.030
Tower rearrangement								
H. Pylori	4.051	-.083	1.280	.154	.498	.530	.937	2.385
Age	-.119***	-.154	-.059	-.121*	-.109**	-.098	-.070	-.130***
H. Pylori x Age	-.049	.004	-.020	-.001	-.007	-.007	-.015	-.035
Symbol-digit substitution								
H. Pylori	-1.073	-.692	.005	-.405	.235	.913	1.616	1.400
Age	-.235***	-.273*	-.239**	-.256***	-.219***	-.165*	-.121	-.233***
H. Pylori x Age	.019	.015	.003	.006	-.004	-.017	-.031*	-.025
Reaction time								
H. Pylori	2.284	-6.638	2.087	5.199	-.280	4.280	5.340	11.212
Age	3.801***	3.390***	3.913***	4.198***	3.780***	4.097***	4.078***	3.782***
H. Pylori x Age	-.035	.130	-.035	-.100	.004	-.076	-.079	-.190
Trails: Numeric								
H. Pylori	132.212	67.624**	13.982	36.929*	12.441	22.965	4.280	99.224*
Age	4.137***	9.577***	4.195**	6.127***	4.117***	4.968***	3.567**	3.197***
H. Pylori x Age	-2.537*	-1.249**	-.250	-.689*	-.226	-.407	-.048	-1.798*
Trails: Alphanumeric								
H. Pylori	194.621	182.426*	13.367	27.287	-13.268	47.443	72.382	153.817

Age	12.886***	31.675***	12.716**	14.311***	10.921***	15.057***	16.437***	11.215***
H. Pylori x Age	-4.186	-3.767**	-.357	-.706	.220	-.896	-1.432	-3.284
Multivariate test[a]								
p	.019	.032	.124	.266	.473	.418	.063	.107

Note: Each model is adjusted for sex, race, education, household income, self-rated health, body-mass index, smoking status, and frequency of drinking alcohol. [a] The multivariate test is a test of the null hypothesis considered within the joint covariance of the dependent variables (i.e., cognitive functioning measures) that age does not moderate the relationship between a measure of *H. pylori* (i.e., *H. pylori* seropositive; the natural log of CagA, VacA, OMP, GroEL, Catalase, and UreA; and the mean of standardized antigens) and cognitive functioning. It is applied here to address potential problems of reporting false negatives because of the number of statistical tests performed. Significant interactions between a H. pylori measure and age are thus ignored if the probability of the multivariate null being true is greater than .05. Numeric N = 796, Reaction time N = 6,757, Reasoning N = 2,269, Pairs matching N = 6,785, Trails numeric N = 394, Trails alphanumeric N = 379, Matrix pattern completion N = 393, Tower rearrangement N = 398. * $p < .05$, ** $p < .01$, *** $p < .001$. Source: *UK Biobank*.

Supplemental Table 2.
Adjusted models of cognitive functioning on the interaction of *H. pylori* and sex: Unstandardized coefficients from linear regression

	Seropositive	ln(CagA)	ln(VacA)	ln(OMP)	ln(GroEL)	ln(Catalase)	ln(UreA)	Mean of standardized antigens
Numeric memory								
H. Pylori	-.060	-.071	-.002	.005	.014	-.040	-.022	-.037
Female	-.368**	-.196	-.319	-.230	-.229	-.486*	-.550**	-.438***
H. Pylori x Female	-.216	-.033	-.031	-.050	-.057	.015	.036	-.098
Reasoning								
H. Pylori	-.447***	-.102*	-.088*	-.105***	-.077***	-.089**	-.062*	-.335***
Female	-.293**	-.492	-.308	-.444*	-.342*	-.319	-.278	-.271**
H. Pylori x Female	.116	.050	.016	.043	.021	.015	.005	.075
Pairs matching incorrect								
H. Pylori	.176	-.089*	-.021	-.010	.021	.007	-.033	-.036
Female	-.014	-.487	-.193	-.204	-.006	-.059	-.178	.025
H. Pylori x Female	.117	.091	.061	.055	.007	.019	.052	.157
Matrix pattern completion								
H. Pylori	.300	-.023	.014	-.043	.008	-.010	-.079	-.094
Female	-.479*	-.676	-.317	-.648	-.451	-.904	-1.278**	-.540**
H. Pylori x Female	-.263	.026	-.074	.019	-.032	.093	.209*	.177
Tower rearrangement								
H. Pylori	.891	.181	.146	.033	-.030	.021	-.017	.111
Female	-1.244**	-.831	-1.334	-1.393	-2.083***	-1.868*	-1.690*	-.889*
H. Pylori x Female	.893	-.071	.096	.096	.321*	.222	.193	.776
Symbol-digit substitution								
H. Pylori	-.293	.054	.143	-.129	-.088	-.215	-.218	-.421
Female	-.099	-.469	-.014	-.538	-.713	-1.520	-.857	.138
H. Pylori x Female	.481	.085	.024	.140	.226	.404	.241	.907
Reaction time								
H. Pylori	2.224	.010	.894	-.560	.360	.427	1.552	1.728
Female	18.621***	11.329	22.395***	15.858**	20.412***	20.613***	22.026***	17.521***
H. Pylori x Female	-3.531	.990	-1.405	.389	-.813	-.773	-1.141	-2.177
Trails: Numeric								
H. Pylori	-16.592	-3.739	-4.772	-2.295	-1.844	.083	2.752	-6.507
Female	-4.010	-29.335	-35.375	-10.336	-11.286	-2.982	9.077	3.723
H. Pylori x Female	19.940	7.202	11.187*	2.984	3.900	1.268	-1.865	16.350
Trails: Alphanumeric								
H. Pylori	-20.616	-21.077	-8.993	-7.550	2.777	-.637	1.758	-13.665
Female	62.865*	64.137	33.189	78.347	85.924*	61.746	95.745	52.424
H. Pylori x Female	-24.271	3.136	7.178	-6.483	-8.492	-.977	-11.453	-18.085

Multivariate test								
p	.607	.787	.692	.594	.027	.865	.432	.371

Note: Each model is adjusted for age, race, education, household income, self-rated health, body-mass index, smoking status, and frequency of drinking alcohol. [a] The multivariate test is a test of the null hypothesis considered within the joint covariance of the dependent variables (i.e., cognitive functioning measures) that sex does not moderate the relationship between a measure of *H. pylori* (i.e., *H. pylori* seropositive; the natural log of CagA, VacA, OMP, GroEL, Catalase, and UreA; and the mean of standardized antigens) and cognitive functioning. It is applied here to address potential problems of reporting false negatives because of the number of statistical tests performed. Significant interactions between a *H. pylori* measure and sex are thus ignored if the probability of the multivariate null being true is greater than .05. Numeric N = 796, Reaction time N = 6,757, Reasoning N = 2,269, Pairs matching N = 6,785, Trails numeric N = 394, Trails alphanumeric N = 379, Matrix pattern completion N = 393, Tower rearrangement N = 398. * $p < .05$, ** $p < .01$, *** $p < .001$. Source: *UK Biobank*.

Supplemental Table 3.
Adjusted models of cognitive functioning on the interaction of *H. pylori* and educational attainment: Unstandardized coefficients from linear regression

	Seropositive	ln(CagA)	ln(VacA)	ln(OMP)	ln(GroEL)	ln(Catalase)	ln(UreA)	Mean of standardized antigens
Numeric memory								
H. Pylori	-.081	-.096*	-.015	.007	-.009	-.022	.016	-.034
College degree	.344**	.295	.315	.581**	.327*	.422	.476*	.266**
H. Pylori x College degree	-.246	.027	-.009	-.078	-.014	-.037	-.053	-.148
Reasoning								
H. Pylori	-.311**	-.060	-.079*	-.068**	-.050**	-.062*	-.030	-.230**
College degree	1.004***	1.249***	.948***	1.074***	1.092***	1.179***	1.227***	.935***
H. Pylori x College degree	-.194	-.036	.002	-.031	-.043	-.058	-.073	-.185
Pairs matching incorrect								
H. Pylori	.246*	-.045	.021	.001	.022	.012	.025	.051
College degree	-.095	-.347	-.030	-.310	-.128	-.158	.168	-.109
H. Pylori x College degree	-.021	.028	-.023	.051	.007	.012	-.078*	-.010
Matrix pattern completion								
H. Pylori	-.241	-.041	-.086	-.078	-.059	.008	.024	-.176
College degree	-.108	-.400	-.323	-.258	-.276	-.120	-.012	.151
H. Pylori x College degree	.857	.079	.127	.088	.111	.062	.033	.379
Tower rearrangement								
H. Pylori	1.432**	.147	.314*	.177	.237**	.064	.061	.679
College degree	.137	-.184	.827	.713	.845	-.568	-.275	.024
H. Pylori x College degree	-.181	-.011	-.239	-.178	-.228	.151	.068	-.353
Symbol-digit substitution								
H. Pylori	.098	.079	.110	.068	.082	-.102	-.226	.002
College degree	.618	.051	.303	1.423	.973	-.221	-.497	.562
H. Pylori x College degree	-.309	.051	.088	-.238	-.122	.199	.298	.100
Reaction time								
H. Pylori	-.810	.109	.149	-.347	.332	.249	.640	.703
College degree	-4.552	-12.880	-3.354	-3.805	.053	-1.271	-6.391	-3.661
H. Pylori x College degree	3.111	1.348	-.091	.013	-1.089	-.618	.767	-.361
Trails: Numeric								
H. Pylori	10.095	5.199	6.804	2.268	1.532	5.498	4.495	15.806
College degree	11.361	67.777	43.055*	24.311	13.025	41.052	22.777	-.729
H. Pylori x College degree	-35.185	-12.499	-12.112*	-5.747	-3.097	-10.049*	-5.783	-30.328*
Trails: Alphanumeric								
H. Pylori	-26.217	-19.326	-11.141	-9.433	-1.703	-5.540	-7.299	-28.248
College degree	19.991	33.530	-18.728	23.498	16.414	-16.816	.263	16.430
H. Pylori x College degree	-13.707	-.256	11.057	-3.170	.650	9.568	5.707	11.924

Multivariate test								
p	.200	.711	.218	.238	.095	.418	.108	.097

Note: Each model is adjusted for age, sex, race, household income, self-rated health, body-mass index, smoking status, and frequency of drinking alcohol. [a] The multivariate test is a test of the null hypothesis considered within the joint covariance of the dependent variables (i.e., cognitive functioning measures) that education does not moderate the relationship between a measure of *H. pylori* (i.e., *H. pylori* seropositive; the natural log of CagA, VacA, OMP, GroEL, Catalase, and UreA; and the mean of standardized antigens) and cognitive functioning. It is applied here to address potential problems of reporting false negatives because of the number of statistical tests performed. Significant interactions between a *H. pylori* measure and education are thus ignored if the probability of the multivariate null being true is greater than .05. Numeric N = 796, Reaction time N = 6,757, Reasoning N = 2,269, Pairs matching N = 6,785, Trails numeric N = 394, Trails alphanumeric N = 379, Matrix pattern completion N = 393, Tower rearrangement N = 398. * $p < .05$, ** $p < .01$, *** $p < .001$. Source: *UK Biobank*.

Supplemental Table 4.

Adjusted models of cognitive functioning on the interaction of *H. pylori* and income (in 10,000 lb.): Unstandardized coefficients from linear regression

	Seropositive	ln(CagA)	ln(VacA)	ln(OMP)	ln(GroEL)	ln(Catalase)	ln(UreA)	Mean of standardized antigens
Numeric memory								
H. Pylori	-.132	-.114*	-.043	.000	-.021	.045	.009	-.028
Income	.023	-.004	.004	.039	.016	.093*	.032	.019
H. Pylori x Income	-.008	.006	.006	-.005	.002	-.019*	-.003	-.013
Reasoning								
H. Pylori	-.528***	-.080	-.052	-.089**	-.079**	-.061	-.086**	-.292**
Income	.081***	.092*	.111***	.082**	.080***	.108**	.069**	.090***
H. Pylori x Income	.032	.002	-.006	.002	.003	-.005	.006	-.001
Pairs matching incorrect								
H. Pylori	.368*	.042	.008	.044	.033	.006	-.030	.066
Income	.002	.095*	-.011	.013	-.001	-.017	-.028	-.008
H. Pylori x Income	-.030	-.018	.001	-.005	-.002	.003	.006	-.004
Matrix pattern completion								
H. Pylori	.659	.007	-.016	.063	.028	.112	.050	.202
Income	.041	.077	.021	.087	.042	.076	.022	.014
H. Pylori x Income	-.096	-.003	-.001	-.018	-.007	-.015	-.002	-.038
Tower rearrangement								
H. Pylori	2.093**	.104	.260	.110	.264*	.109	-.069	.592
Income	.075	.093	.086	.066	.128	.032	-.069	.043
H. Pylori x Income	-.145	.007	-.012	-.005	-.025	.006	.031	-.014
Symbol-digit substitution								
H. Pylori	1.283	.284	.700*	.128	.379*	.350	-.267	1.007
Income	.210*	.274	.465**	.270	.376**	.422*	.007	.125
H. Pylori x Income	-.259	-.035	-.099*	-.034	-.068*	-.072	.035	-.179
Reaction time								
H. Pylori	-2.562	-.160	-.466	-1.352	-.106	-1.187	1.107	-1.404
Income	-2.271***	-2.669	-2.532*	-2.992***	-2.114**	-3.196**	-1.936*	-2.037***
H. Pylori x Income	.676	.176	.134	.228	.008	.289	-.039	.462
Trails: Numeric								
H. Pylori	-25.259	-15.848*	.984	-7.673	-3.714	-2.804	4.870	-9.412
Income	-2.790	-15.951**	-1.697	-6.888*	-4.504	-4.663	.352	-1.746
H. Pylori x Income	3.658	3.020*	-.055	1.300	.749	.717	-.599	2.121
Trails: Alphanumeric								
H. Pylori	-62.442	-63.309**	-20.791	-28.267*	-3.400	-8.935	3.897	-54.191
Income	-11.808*	-57.271**	-19.617*	-22.693*	-11.916	-16.926	-4.596	-9.892*
H. Pylori x Income	5.723	8.165*	2.756	3.181	.377	1.569	-1.574	5.776

Multivariate test								
p	.562	.091	.219	.281	.308	.148	.672	.614

Note: Each model is adjusted for age, sex, race, education, self-rated health, body-mass index, smoking status, and frequency of drinking alcohol. [a] The multivariate test is a test of the null hypothesis considered within the joint covariance of the dependent variables (i.e., cognitive functioning measures) that age does not moderate the relationship between a measure of *H. pylori* (i.e., *H. pylori* seropositive; the natural log of CagA, VacA, OMP, GroEL, Catalase, and UreA; and the mean of standardized antigens) and cognitive functioning. It is applied here to address potential problems of reporting false negatives because of the number of statistical tests performed. Significant interactions between a *H. pylori* measure and age are thus ignored if the probability of the multivariate null being true is greater than .05. Numeric N = 796, Reaction time N = 6,757, Reasoning N = 2,269, Pairs matching N = 6,785, Trails numeric N = 394, Trails alphanumeric N = 379, Matrix pattern completion N = 393, Tower rearrangement N = 398. * $p < .05$, ** $p < .01$, *** $p < .001$. Source: *UK Biobank*.
